# Supplementary material for: Is Adherence to the Saudi Healthy Plate Dietary Guidelines Associated with Eating Concerns Symptoms among Saudi Young Females?
Source: Nutrients. 2024 Jun 18;16(12):1931. doi: 10.3390/nu16121931 (PMC11206548; doi:10.3390/nu16121931)
Supplement: Supplementary file 1 [file nutrients-16-01931-s001.zip › nutrients-3047539-supplementary.pdf]

Table S1: The Saudi Healthy Plate Dietary Guidelines (SHPDGs) Scores.

Food Groups	Options	Score
Fruits & Vegetables / per day (e.g., Fresh, Dried, Juice, Cooked)	≥5 Serving*	0
	3–4 Serving	1
	1–2 Serving	2
	Never	2
Grains & Bread / per day (e.g., Bread, Toast, Sandwich, Rice, Pasta, Oats, Corn flacks)	3–4 Serving*	0
	1–2 Serving	1
	Never	2
Milk & Dairy Products / per day (e.g., Milk, Laban, Yogurt, Cheses, Labneh, Cream)	≥3 Serving*	0
	2 Serving	1
	1 Serving	2
	Never	2
Meat & Substitutes / per day (e.g., Sheep, Fish, Chicken, Egg, Legumes)	≥3 Serving*	0
	2 Serving	1
	1 Serving	2
	Never	2
Fats / per day (e.g., Butter, Ghee, Oil)	Very little*[a]	0
	Some/Appropriate amount	1
	A lot	2
Water / per day	≥6 Cups*[b]	0
	5–6 Cups	1
	2–4 Cups	2
	<2 Cups	2
Fast Foods / per week (e.g., Burger, Pizza)	Never*[c]	0
	1–3 Times	1
	≥4 Times	2
Soft Drinks / per week	Never*[c]	0
	1–2 Times	1
	≥3 Times	2
Crackers & Crisps / per week (e.g., Chips, Biscuit, Pretzel)	Never*[c]	0
	1 Time	1
	1–3 Times	2
	≥4 Times	2
Sweet & Dessert / per week (e.g., Chocolate, Donut, Basbousa, Candy)	Never*[c]	0
	1–3 Times	1
	≥4 Times	2
Physical Activity / per week	Daily*[c]	0
	3–4 Times weekly	1
	Never	2

* Recommended by the Saudi Healthy Plate Dietary Guidelines; [a] Done by (Paxton et al., 2011), [b] One cup equivalent to 200 ml; [c] Suggested by the Authors.
